# Fine-scale genetic structure of the European bitterling at the intersection of three major European watersheds

**DOI:** 10.1186/s12862-018-1219-9

**Published:** 2018-07-04

**Authors:** Veronika Bartáková, Josef Bryja, Martin Reichard

**Affiliations:** 1 The Czech Academy of Sciences, Institute of Vertebrate Biology, Květná 8, 603 65 Brno, Czech Republic; 2 Department of Botany and Zoology, Faculty of Science, Masaryk University, Kotlářská 2, 611 37 Brno, Czech Republic

**Keywords:** Cryptic invasions, Freshwater conservation, Game fish stocking, Gene flow, Human-mediated translocation, Phylogeography, Population genetics

## Abstract

**Background:**

Anthropogenic factors can have a major impact on the contemporary distribution of intraspecific genetic diversity. Many freshwater fishes have finely structured and locally adapted populations, but their natural genetic structure can be affected by river engineering schemes across river basins, fish transfers in aquaculture industry and conservation management. The European bitterling (*Rhodeus amarus*) is a small fish that is a brood parasite of freshwater mussels and is widespread across continental Europe. Its range recently expanded, following sharp declines in the 1970s and 1980s. We investigated its genetic variability and spatial structure at the centre of its distribution at the boundary of three watersheds, testing the role of natural and anthropogenic factors in its genetic structure.

**Results:**

Sequences of mitochondrial cytochrome B (*CYTB*) revealed that bitterling colonised central Europe from two Ponto-Caspian refugia, which partly defines its contemporary genetic structure. Twelve polymorphic microsatellite loci revealed pronounced interpopulation differentiation, with significant small-scale differentiation within the same river basins. At a large scale, populations from the Baltic Sea watershed (middle Oder and Vistula basins) were distinct from those from the Black Sea watershed (Danube basin), while populations from rivers of the North Sea watershed (Rhine, Elbe) originated from the admixture of both original sources. Notable exceptions demonstrated the potential role of human translocations across watersheds, with the upper River Oder (Baltic watershed) inhabited by fish from the Danube basin (Black Sea watershed) and a population in the southern part of the River Elbe (North Sea watershed) basin possessing a signal of admixture from the Danube basin.

**Conclusions:**

Hydrography and physical barriers to dispersal are only partly reflected in the genetic structure of the European bitterling at the intersection of three major watersheds in central Europe. Drainage boundaries have been obscured by human-mediated translocations, likely related to common carp, *Cyprinus carpio*, cultivation and game-fish management. Despite these translocations, populations of bitterling are significantly structured by genetic drift, possibly reinforced by its low dispersal ability. Overall, the impact of anthropogenic factors on the genetic structure of the bitterling populations in central Europe is limited.

**Electronic supplementary material:**

The online version of this article (10.1186/s12862-018-1219-9) contains supplementary material, which is available to authorized users.

## Background

Quaternary climatic changes have significantly affected the genetic diversity and structure of populations. The current interspecific and intraspecific diversity of the European biota has been substantially shaped by cycles of expansion and contraction of species ranges (e.g. [1]). Thermophilic species repeatedly retreated from much of their continental ranges during cold glacial periods, followed by range expansions and recolonisations during warmer periods [2–4]. Similar periodic range expansions and contractions due to climatic fluctuations occurred over much shorter timespans within the Holocene [5]. Such dynamics frequently generated temporarily isolated populations in local refugia. Such isolated populations tended to differentiate due to genetic drift and local adaptation. Consequent (re)colonization processes are associated with reduced genetic diversity due to sequential founder effects and population bottlenecks [6]. When contemporary ranges were recolonized from multiple refugia, distinct genetic lineages sometimes met in contact zones and hybridized (e.g. [7, 8]), thereby increasing genetic diversity in these secondary contact zones.

The geographic patterns and genetic diversity hotspots of freshwater taxa often differ from those of terrestrial taxa. The historical dynamics of range contractions, expansions and shifts in freshwater species are less well understood than those of terrestrial taxa [9]. Freshwater systems support species and populations that are often more isolated than terrestrial taxa, depending on their ability to overcome natural barriers to dispersal. For example, salt-tolerant and migratory species [10, 11] display shallow genetic divergence and high levels of gene flow that are not reflected in terrestrial species. In contrast, freshwater organisms often display a high level of genetic differentiation among populations [12, 13]. In these taxa, natural dispersal and colonisations may be limited to relatively rare events, such as extensive pluvial periods, river captures, large-scale flooding and historical connections of currently separated rivers during periods of reduced sea level (reviewed in [14]).

Human-mediated translocations are an important recent influence on species distributions and population connectivity [15–17], particularly for freshwater ecosystems where connections were previously rare. For example, artificial canals can connect previously separated river basins [18], species are unintentionally transferred across drainages [19], and angling-related fish transfers [20] distribute both game and bait species outside their former geographic ranges [15] and thereby mix previously isolated populations [21]. Other human activities, such as aquaculture, release of non-native species, river regulation and channelization, all may affect the population genetics of native species [22–24].

Here, we investigate fine-scale population genetic structure of a small freshwater fish, the European bitterling, *Rhodeus amarus* Bloch (Cyprinidae) in the central part of its current distribution*.* The bitterling is a thermophilic species currently distributed across much of continental Europe, excluding Fennoscandia, the Iberian peninsula, and northern parts of European Russia [25–29]. This species has a limited dispersal capacity [30] and no commercial value [31], limiting its potential for human-assisted dispersal. However, the bitterling benefits from river regulation and channelization [32] and uses newly constructed waterways and connections between drainages to colonise new areas [25]. Human-assisted translocations were also reported, and the European bitterling has been successfully introduced to England [33], Italy [34] and Denmark [35]. The European bitterling was formerly kept as an ornamental fish, but its current popularity is negligible [26]. Bitterling lay their eggs into the gill chambers of living mussels [36], making the extent of its distribution contingent upon the presence of freshwater mussels (families Unionidae and Margaritiferidae).

The current wide distribution of the bitterling across Europe contrasts with a marked decline in its abundance in the 1970s and 1980s that led to its designation as an endangered species in several European countries [26, 37]. While a decrease in water quality and declines in populations of unionid mussels were originally considered the primary reasons for the reduction in its abundance, a relatively cold climatic period also may have contributed to its decline. Using historical bibliographic records, Van Damme et al. [26] argued that the bitterling colonised much of Europe during the Medieval Warm period, disappeared from most of continental Europe during the Little Ice Age (approximately 1600–1850 AD) and expanded again from isolated populations following a subsequent warming of European climate.

Previous phylogeographic studies demonstrated that the European bitterling persisted during colder Quaternary conditions in southern refugia and colonised the continent from two main regions [27–29]. First, the lower part of the Danube was the refuge for the “Western lineage” that colonised the entire Danube basin and western Europe (the Rhine and Elbe basins) [27]. Second, a region north of the Black Sea was the refuge for the “Eastern lineage” that colonized north-eastern Europe, including areas east of the River Vistula in central Poland (including the Rivers Dnieper and Dniester in the Ukraine and European part of Russia) [27]. Using nuclear microsatellite markers, Bryja et al. [29] demonstrated admixture between the two main lineages in their contact zones in central and western Europe. Natural contact and admixture between the lineages was possible during dynamic changes to river drainage systems [38, 39], although unintentional introductions related to the onset of common carp (*Cyprinus carpio* L.) aquaculture in western Europe 5–10 centuries ago also are possible [26, 29]. Approximate Bayesian Computation based on microsatellite markers suggested that bitterling populations from western Europe (the Rhine, the Rhône) are of Holocene origin (median estimate of their origin 8000 years ago), with secondary contact in the Elbe basin approximately 1500 (range: 212–3729) years ago [29]. Other refugial populations did not contribute to current bitterling repopulation of Europe and remained confined to the eastern Mediterranean region [28, 29, 31, 40, 41].

**Fig. 1:**
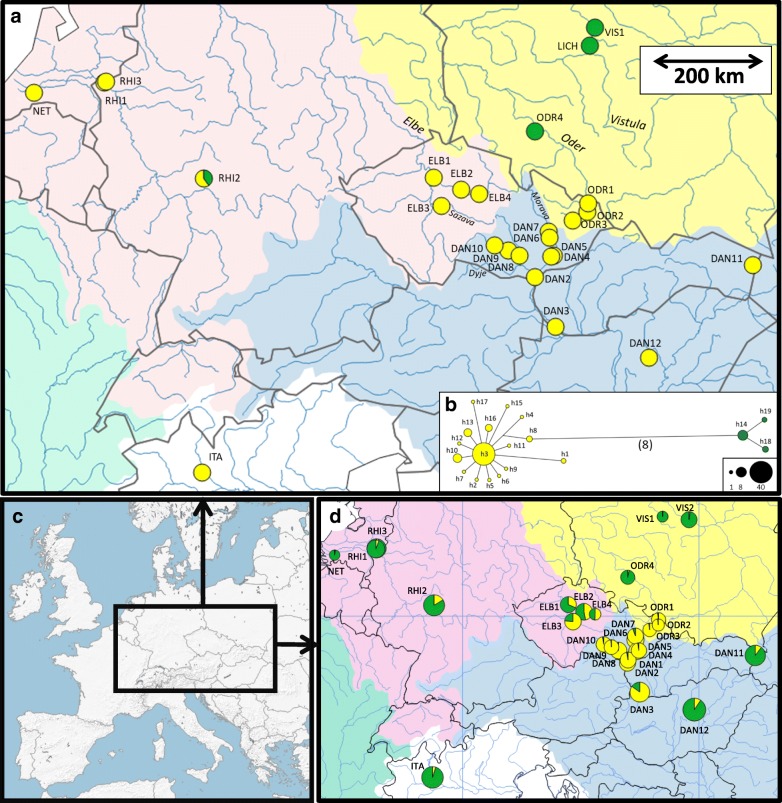
Geographic distribution of genetic diversity of mitochondrial and microsatellite markers. **a** Spatial distribution of two main mtDNA lineages, based on **b** median-joining haplotype network of 83 *CYTB* sequences (1124 bp) from 26 populations of the European bitterling. Length of branches in the network is proportional to the number of substitutions along a given branch (the number of substitutions in brackets for the main division), and circle size is proportional to haplotype frequency (reference scale provided). Colours of portions of pie-charts indicate the relative proportions of haplogroups at particular locations. **c** Overall geographic setting of the study area on the map of Europe, **d** microsatellite marker-based genetic structure of bitterling populations at the border of three watersheds. Colours of portions of in pie charts correspond with the inferred membership of individuals to a particular group *K* detected in STRUCTURE for *K* = 2; size of pie charts indicates allelic richness. River basins are illustrated using different colours. The map was created in QGIS 2.18 (http://qgis.org)

In the present study, we focused on fine-scale patterns of genetic structure in the central region of the current distribution of the European bitterling that encompasses the main European watershed divides between three major basins. We tested the roles of hydrography and human activities on the present genetic diversity of bitterling, which is a legally protected but widely distributed species [31]. Specifically, we tested the roles of natural and human-assisted dispersal by spatial analysis of detailed genetic structure using a mitochondrial marker (the gene for cytochrome *b*; *CYTB*) and a set of 12 nuclear microsatellite markers. We concentrated sampling in the Czech Republic, where the main divides among three major European watersheds exist (Fig. 1), using data from a combination of three previously genotyped populations [29] and 13 new populations genotyped for this study. The Black Sea basin includes the River Morava (a northern tributary of the Danube) and its tributaries in the south-eastern part of the country. The North Sea basin includes the Elbe basin in the western Czech Republic. The Baltic Sea basin includes the River Oder basin in the north-eastern part of the country. We expanded the study area to genotype populations in neighbouring countries that were not included in our previous range-wide population genetic study [29] and were potentially informative for our aims.

The specific aims of the study were: (1) to test whether the genetic structure of the bitterling in central Europe is contingent upon the boundary of three major watersheds, and (2) to compare the roles of natural and anthropogenic processes in formation of the contemporary genetic structure in the study area. In broader terms, our aim was to contribute to understanding of the impacts of artificial translocations, historical events and demographic processes in shaping present genetic diversity of freshwater organisms using a non-commercial fish in a hydrographically diverse area.

## Material and methods

### Sampling and genotyping

In total, we analysed 691 samples of fish (small tissue clips from caudal fins stored in 96% ethanol) from 28 populations, composed of 409 newly sampled and genotyped individuals and 282 individuals from a previous study [29]. We focused on the boundary between three different watersheds (Baltic, North and Black Sea drainages) in central Europe (Fig. 1 and Table 1). Fish were collected during 2003–2009 using electrofishing as part of routine monitoring of fish communities, or provided by collaborators from Germany, Poland, the Netherlands and Hungary (Table 1). We aimed at a sample size of 25 individuals per population, although this number could not be achieved in several populations despite high sampling effort (Table 1). All bitterling individuals were genotyped at 12 microsatellite loci [42, 43] and a subset of individuals were sequenced at the mitochondrial gene cytochrome *b* (*CYTB*) gene (see Additional file 1 for more details).

**Table 1:** Overview of analysed populations

Pop ID	Locality	Country	Latitude	Longitude	*N*_total_	*N*_CYTB_	Basin^a^
DAN1*	Kyjovka River	Czech Rep.	N 48°46’48”	E 17°00’58”	45	–	Danube (Dyje)
DAN2*	Morava River floodplain	Czech Rep.	N 48°41’35”	E 16°59”56”	34	1	Danube (Morava)
DAN3*	Gabčíkovo, Priehradky	Slovakia	N 47°53’00”	E 17°30’00”	15	4	Danube
DAN4	Morava River near Nedakonice	Czech Rep.	N 49°01’45”	E 17°23’33”	24	2	Danube (Morava)
DAN5	Olšava River near Kunovice	Czech Rep.	N 49°02’44”	E 17°28’04”	24	2	Danube (Morava)
DAN6	Haná River near Bezměrov	Czech Rep.	N 49°20’23”	E 17°20’41”	25	2	Danube (Morava)
DAN7	Bečva River near Troubky	Czech Rep.	N 49°26’00”	E 17°20’19”	23	2	Danube (Morava)
DAN8	Litava River near Židlochovice	Czech Rep.	N 49°02’29”	E 16°36’57”	24	2	Danube (Dyje)
DAN9	Oslava River near Oslavany	Czech Rep.	N 49°07’37”	E 16°19’47”	9	2	Danube (Dyje)
DAN10	Jihlava River near Vladislav	Czech Rep.	N 49°12’33”	E 15°59’29”	23	2	Danube (Dyje)
DAN11*	River Ublianka at Ubla	Slovakia	N 48°53’56”	E 22°23’26”	21	5	Danube (Tisza)
DAN12*	Tápio stream, near Tápiószele	Hungary	N 47°21’49”	E 19°49’22”	25	5	Danube (Tisza)
RHI1*	Grietherother Altrhein, NW of Rees	Germany	N 51°47’17”	E 06°22’17”	27	5	Rhine
RHI2	Nida River	Germany	N 50°17’00”	E 08°47’47”	25	5	Rhine
RHI3	Lower Rhine	Germany	N 51°47’06”	E 06°20’04”	22	3	Rhine
NET*	Mark, Zevenbergen	Netherlands	N 51°37’34”	E 04°35’08”	16	5	Rhine
ELB1*	Labe River near Obříství	Czech Rep.	N 50°17’48”	E 14°28’53”	46	3	Elbe
ELB2	Labe River near Libický luh	Czech Rep.	N 50°06’05”	E 15°09’28”	22	3	Elbe
ELB3	Sázava River at Poříčí n. S.	Czech Rep.	N 49°50’27”	E 14°40’51”	26	3	Elbe
ELB4	Labe River, at Valy	Czech Rep.	N 50°01’59”	E 15°36’58”	18	3	Elbe
ODR1	Oder River at Bohumín	Czech Rep.	N 49°53’25”	E 18°18’24”	36	3	Oder
ODR2	ponds near Paskov - Pilíky	Czech Rep.	N 49°45’12”	E 18°16’54”	27	3	Oder
ODR3	River Oder at Jeseník n. O.	Czech Rep.	N 49°37’01”	E 17°55’11”	22	3	Oder
ODR4	River Sleza at Wroclaw	Poland	N 51°01’51”	E 16°59’55”	22	5	Oder
VIS1*	Lake Kociolek	Poland	N 52°37’02”	E 18°28’42”	29	3	Vistula
VIS2*	Wloclawski reservoir	Poland	N 52°33’00”	E 19°35’00”	24	–	Vistula
ITA	Italy, introduction	Italy	N 45°24’09”	E 08°44’25”	34	4	Po
LICH	Lake Licheńskie	Poland	N 52°20’22”	E 18°21’28”	(3)	3	Oder

*N*_total_ = the number of individuals genotyped at 12 microsatellites; *N*_CYTB_ = a subset of individuals genotyped for partial cytochrome *b* sequences. Three individuals from the LICH population were genotyped only for *CYTB* sequences (*N* total given in brackets). Populations labelled * were genotyped by Bryja et al. [29]. ^a^Subbasins are shown for the Danube basin in parentheses

### Genetic structure based on microsatellites and mtDNA

A Hardy-Weinberg equilibrium (*HWE*) test was performed for each locus in all populations using Markov chain methods (“Exact probability test”) in Genepop 4.0.10 [44]. Genetic variability was estimated for each locus in each population from expected (*H*_*e*_) and observed (*H*_*o*_) heterozygosities in GENETIX 4.05 [45]. Because of dependence of the detected number of alleles on sample size, we calculated allelic richness (*AR*) corrected for minimum sample size (*N* = 9) using the rarefaction method in FSTAT 2.9.3.2 [46]. The proportion of null alleles (*NA*) at each locus and population was estimated in FreeNA [47]. The probability of the presence of null alleles, allele dropout and scoring errors due to stutter was further tested using MICRO-CHECKER 2.2.3 [48].

Genetic divergence among study populations was estimated by pairwise *F*_ST_ [49], and the presence of substructure (i.e., *F*_ST_ significantly higher than zero) was tested by 1000 permutations in GENETIX 4.05 [45]. We analysed the population genetic structure of microsatellite genotypes using the Bayesian clustering algorithm implemented in the program STRUCTURE 2.3.3 [50]. The computation for *K* = 1–10 was completed using an admixture model and correlated allele frequencies model (λ = 1). The program was run with 10 independent simulations for each value of *K*, each of 10^6^ iterations, following a burn-in period of 10^5^ iterations. The likelihood of *K* (Ln Pr(X|K)), was used to infer the best number of real populations in the datasets (Additional file 2: Figure S1a) in combination with the estimation of the best *K* based on the Δ*K* criterion using the method of Evanno et al. [51] (Additional file 2: Figure S1b). The results of 10 replicates for each value of *K* were combined using the Greedy algorithm in CLUMPP 1.1.2 [52], and summary for each *K* were visualised using Distruct v. 1.1 [53]. In addition to individual-based clustering, genetic relationships among populations based on allele frequencies were also assessed by factorial correspondence analysis (FCA) in GENETIX 4.05, and the positions of each population on the first two axes were graphically illustrated. The sequence variation in *CYTB* was visualised using the median-joining algorithm in Network 4.610 as a haplotype network [54]. All sequences were geo-referenced and the geographical distribution of haplogroups was inspected visually.

Isolation by distance was analysed for the group of Danube populations (DAN1-DAN10) by regressing pairwise estimates of *F*_ST_/(1- *F*_ST_) against ln-distance between sample sites [55] measured along hydrographic distances (river kilometers). Mantel tests were used to test the correlation between matrices of genetic differentiation and Euclidean distances between sampling sites by 1000 permutations in GENEPOP.

### Specific tests of the role of barriers among three watersheds

Hierarchical Analysis of Molecular Variance (AMOVA; [56]), based on allele frequencies at microsatellites, was carried out using Arlequin 3.1 [57]. Populations close to watershed divides were grouped according to the three river basins – Elbe (ELB1-ELB4), Danube (DAN1, DAN2, DAN4-DAN10), and Oder (ODR1-ODR4). The first test considered all these populations and tested the role of watersheds on the genetic structure. In the second test, we removed four populations that were identified to be either admixed (ELB3) or assigned to unexpected groups (ODR1-ODR3) in the STRUCTURE analysis. The procedure examined the effect of these four populations on the strength of watershed-based genetic structure. The Fixation Index (*F*_*CT*_) was calculated, and the significance of partitioning of molecular variance among groups was assessed by 10,000 bootstraps [56].

To examine temporal aspects of the observed divergence, we compared the relative impact of genetic drift (*F*_*ST*_) and stepwise mutations (*R*_*ST*_) on genetic differentiation in SPAGeDi v1.5 [58], where *F*_ST_ is a measure of population differentiation [49], *R*_*ST*_ is an *F*_*ST*_ analogue based on allele size, and *pR*_ST_ is *R*_ST_ computed after allele-size permutation. The hypothesis *R*_*ST*_ = *F*_*ST*_ predicted that genetic drift had an equal effect on genetic divergence as stepwise mutations. The alternative hypothesis (*R*_*ST*_ > *F*_*ST*_) predicted that stepwise mutations were more heavily involved in population differentiation [59], suggesting older divergence. Different allele sizes at each locus were randomly permuted among allelic states, and 9000 random permutations provided a simulated distribution of *R*_*ST*_ values (*pR*_*ST*_) and their 95% confidence intervals (CIs) for testing whether *R*_*ST*_ > *pR*_*ST*_ [59]. The analysis was based on data from all 12 loci.

### Scenarios of population history assessed by approximate Bayesian computation

The analyses of genetic structure revealed that some populations (sample ELB3: Sázava River (SAZAVA); and samples ODR1-ODR3: the Czech part of the River Oder (CZODER), see above) did not cluster according to watersheds. We inferred their detailed history via Approximate Bayesian Computation (ABC; [60]) implemented in DIYABC 2.0.4 [61]. This program enables modelling of complex population histories with any combination of population divergences, admixtures and population size fluctuations. It allows comparison of alternative evolutionary scenarios, estimation of their relative support, and quantification of parameters for particular scenarios [61]. The ABC analysis is based on modelling of population history and both mutations (using generalized stepwise mutation model) and genetic drift (by specification of effective population sizes; for example modelling founder effect by a strong decrease of *N*_e_) are taken into account. We acknowledge, however, that an alternative view (hold over several revisions by an anonymous reviewer) might be that the ABC analysis rests on the assumption that differences among populations are solely due to mutations.

Three groups of populations were created according to the Bayesian assignment of their genetic structure for testing the origin of hydrographically misclassified populations. First, SAZAVA (North Sea) was formed by the ELB3 population (26 individuals), the Elbe group (CZELBE – North Sea) composed of ELB1, ELB2, ELB4 (86 individuals), and a group of geographically proximate populations associated with western tributaries of the River Morava (WMORAV – Black Sea) consisting of populations DAN8-DAN10 (56 individuals). Second, the Czech Oder populations (CZODER – Baltic Sea) consisted of ODR1–3 (85 individuals), the River Oder and Vistula populations from Poland (POLRIV - Baltic Sea) comprising ODR4, VIS1, VIS2 (75 individuals), and populations from geographically close rivers in the lower and middle reaches of the River Morava and its tributaries (NMORAV – Black Sea) formed by DAN1, DAN2, and DAN4-DAN7 (175 individuals).

Nine scenarios were constructed to estimate the most likely description of the observed pattern of population structuring among watersheds (Additional file 3: Table S1, Figures S1, S2). Effective population sizes, timing of events (merging, splitting or changes in effective population size), and rates of admixture in the case of merging events were used to describe the scenarios. The range of uniform priors is specified in Table S2 (Additional file 3). All markers had regular motifs (motif length of 4 bp for *Rser11* and 2 bp in all other markers used), and the generalized stepwise model was used as the mutation model (GSM; [62]). All microsatellite mutation parameters were at default settings. We used a generation time of one year [36, 63].

We simulated 1 million data sets per scenario. For each simulation, a set of summary statistics was computed for comparison to the observed data set for selection of the best model. Logistic regression was used to select among models. The relative posterior probability (95% credible intervals) of each scenario was determined with the 1% of the simulated data sets closest to the observed data (Euclidian distances). The posterior parameter distributions were estimated from the 1% of the simulated data sets closest to the most likely scenario [64]. Model checking was performed to evaluate the discrepancy between a model-posterior combination and observed data set by considering sets of summary statistics that had not been used for previous inferential steps.

## Results

Genotyping success for microsatellites was high (96.1%), and multilocus genotypes were obtained for 688 individuals from 27 populations. Most missing genotypes were at loci *Rser04* (7.8%) and *Rser09* (7.3%), possibly representing homozygotes for null alleles. The same loci had relatively high frequencies of null alleles estimated in FreeNA (6.9% and 7.9%, respectively, Additional file 1: Table S1), despite no apparent evidence of null alleles, allele dropout or scoring errors due to stuttering in results assessed by MICRO-CHECKER. Further, the *Rser04* and *Rser13* loci possessed a high level of polymorphism (94 and 95 alleles) (Additional file 1: Table S1) with large differences among populations (1–31 and 7–35 alleles per population for *Rser04* and *Rser13*, respectively). To avoid potential bias caused by these loci, population-level genetic variability was additionally analysed using a reduced dataset of 9 loci (i.e., excluding loci *Rser04*, *Rser09* and *Rser13*). All analyses of genetic structure among populations are based on the complete dataset across all 12 loci because high allelic polymorphism and presence of null alleles had a negligible effect on the analyses of population structure. The analysis of mitochondrial variability is based on 83 *CYTB* sequences from 26 populations.

### Intra-population genetic diversity

Departure from *HWE* was demonstrated in 41% of populations (11 of 27) when calculated over all 12 loci, although only a single population deviated from the *HWE* in the reduced dataset with 9 loci (i.e., after removing loci with null alleles). This population (ITA) arose from a recent introduction [65], and deviation from *HWE* (deficit of heterozygotes) may be due to inbreeding or to *HWE* not yet having been achieved. The range of allelic richness (*AR*) for 9 loci was 2.33–5.05 (rarefaction estimate for 9 individuals, Additional file 2: Figure S2). All measures of intrapopulation genetic variation (*H*_*o*_, *H*_*e*_, and *AR* for 9 and 12 loci) were strongly correlated (*p* < 0.05; Additional file 2: Table S1).

The lowest genetic diversities were in populations at the north-western periphery of the distribution of *R. amarus* (NET, VIS1), one Czech population in the River Elbe (ELB4), and two populations in the Czech part of the River Oder (ODR2, ODR3). The highest genetic diversities were detected in the middle part of the Danube basin (DAN3, DAN11, DAN12; all outside the Czech Republic), an apparently admixed population from the River Rhine basin (RHI2; see below) and, surprisingly, an introduced Italian population (ITA) (Additional file 2: Table S1, Additional file 2: Figure S2). The highest genetic diversities within the Czech Republic were detected in two populations in the Morava basin (DAN4, DAN6), one population in the Dyje basin (DAN8) and in two Elbe populations (ELB2, ELB3).

### Spatial genetic structure

The mitochondrial *CYTB* haplotype network revealed two main lineages corresponding with the western and eastern lineages sensu Bohlen et al. [27] (Fig. 1). The western lineage exclusively dominated all three watersheds in the Czech Republic. The eastern mitochondrial lineage was detected in the River Vistula and middle River Oder in Poland (Baltic watershed). The two lineages co-occurred in the River Nida (RHI2), the River Rhine tributary (North Sea watershed); two individuals from the RHI2 population possessed eastern lineage haplotypes (h14), while three individuals had western-lineage haplotypes (h3, h17). The western haplogroup demonstrated a star-like haplotype network (Fig. 1b), indicating a recent demographic expansion in central Europe.

We detected a high level of genetic structuring and inferred a significant role of genetic drift. The pairwise *F*_ST_ values were significantly different from zero in 98.9% of population pairs (Table 2). The *F*_ST_ indicated low differentiation between only two geographically neighbouring populations from the Rivers Kyjovka (DAN1) and lower Morava (DAN2), and among three populations in the middle reaches of the River Morava (DAN4) and its geographically proximate tributaries, the Rivers Bečva (DAN7) and Olšava (DAN5). The genetic distance between Danube basin populations was significantly correlated with their hydrographic distance (Mantel test, 1000 permutations, *p* = 0.001), suggesting a strong role of isolation by distance.

**Table 2:** Matrix of pairwise *F*_ST_ values (above the diagonal) and pairwise *R*_ST_ values (below the diagonal)

	DAN1	DAN2	DAN3	DAN7	DAN8	DAN10	DAN11	DAN12	DAN4	DAN6	DAN9	DAN5	RHI1	RHI2	RHI3	NET	ELB1	ELB2	ELB3	ELB4	ODR1	ODR2	ODR3	ODR4	VIS1	VIS2	ITA
DAN1		0.005*	0.045	0.034	0.072	0.056	0.195	0.18	0.029	0.056	0.113	0.02	0.159	0.131	0.158	0.263	0.136	0.112	0.082	0.147	0.074	0.171	0.091	0.33	0.466	0.399	0.184
DAN2	−0.009		0.05	0.031	0.08	0.049	0.213	0.198	0.026	0.053	0.098	0.023	0.165	0.144	0.163	0.28	0.143	0.127	0.08	0.159	0.088	0.189	0.103	0.351	0.494	0.414	0.195
DAN3	0.072	0.058		0.07	0.06	0.068	0.155	0.143	0.062	0.104	0.081	0.075	0.108	0.085	0.122	0.209	0.118	0.107	0.068	0.156	0.074	0.137	0.082	0.318	0.481	0.392	0.147
DAN7	0.027	0.008	0.044		0.05	0.028	0.217	0.199	0.003*	**0.031**	0.089	0.002*	0.171	0.14	0.174	0.301	0.145	0.108	0.069	0.154	0.092	0.179	0.106	0.355	0.522	0.421	0.197
DAN8	0.126	0.099	0.057	0.019		0.046	0.174	0.16	0.059	0.093	0.053	0.063	0.145	0.113	0.147	0.272	0.151	0.109	0.074	0.151	0.079	0.162	0.094	0.348	0.493	0.397	0.165
DAN10	0.081	0.082	0.272	0.121	0.295*		0.206	0.193	0.03	0.082	0.051	0.05	0.165	0.135	0.17	0.294	0.139	0.109	0.045	0.142	0.103	0.187	0.114	0.354	0.504	0.41	0.203
DAN11	0.103	0.108	0.334*	0.165	0.371	0.004		0.029	0.194	0.202	0.238	0.215	0.098	0.101	0.093	0.288	0.229	0.199	0.203	0.224	0.215	0.289	0.224	0.375	0.506	0.412	0.063
DAN12	0.132	0.139	0.397*	0.197	0.413*	0.02	−0.021		0.171	0.183	0.218	0.197	0.06	0.075	0.057	0.239	0.195	0.163	0.172	0.202	0.198	0.252	0.201	0.312	0.438	0.347	0.035
DAN4	0.018	0.004	0.110	−0.013	0.092	0.062	0.107	0.134		**0.013**	0.09	0.001*	0.139	0.11	0.143	0.269	0.118	0.099	0.042	0.155	0.089	0.171	0.1	0.313	0.476	0.372	0.168
DAN6	0.002	−0.01	0.068	−0.013	0.075	0.076	0.114	0.141	−0.017		0.124	**0.016**	0.16	0.136	0.159	0.297	0.152	0.127	0.087	0.173	0.124	0.194	0.15	0.326	0.487	0.383	0.178
DAN9	0.049	0.039	0.207	0.038	0.206	−0.007	0.051	0.083	−0.004	0.018		0.103	0.181	0.152	0.187	0.333	0.176	0.165	0.08	0.214	0.14	0.193	0.155	0.392	0.56	0.441	0.214
DAN5	0.008	−0.006	0.064	−0.025	0.055	0.061	0.087	0.108	−0.029	−0.024	−0.009		0.166	0.139	0.167	0.301	0.15	0.122	0.082	0.167	0.084	0.178	0.105	0.349	0.514	0.413	0.189
RHI1	0.094	0.109	0.234*	0.188	0.341*	0.123	0.085	0.131*	0.169	0.143	0.158	0.139		0.035	0.008	0.118	0.103	0.116	0.125	0.168	0.176	0.21	0.176	0.204	0.331	0.238	0.068
RHI2	0.029	0.012	0.088	−0.016	0.068	0.129	0.162*	0.191*	−0.004	−0.01	0.058	−0.021	0.185*		0.041	0.121	0.079	0.075	0.087	0.13	0.147	0.189	0.154	0.162	0.294	0.201	0.076
RHI3	−0.003	−0.005	0.094	0.025	0.165	0.117	0.124	0.171*	0.028	0.007	0.092	−0.001	0.086*	0.019		0.164	0.125	0.133	0.135	0.177	0.18	0.234	0.179	0.237	0.371	0.257	0.058
NET	0.024	0.012	0.064	0.001	0.079	0.106	0.116	0.144	0.013	0.001	0.052	−0.011	0.115	−0.015	0.000		0.121	0.177	0.231	0.25	0.303	0.329	0.309	0.192	0.313	0.252	0.247
ELB1	0.132	0.126	0.352*	0.133	0.308	0.031	0.091	0.092	0.068	0.101	0.000	0.072	0.252	0.136	0.183	0.128		0.053	0.091	0.099	0.18	0.21	0.179	0.128	0.248	0.196	0.203
ELB2	0.099	0.078	0.216	0.027	0.142	0.092	0.147	0.161	0.012	0.04	0.011	0.007	0.246*	0.040	0.123	0.047	0.038		0.082	0.046	0.147	0.198	0.153	0.189	0.312	0.251	0.178
ELB3	0.118	0.121	0.335*	0.166	0.358*	−0.021	0.017	0.033	0.099	0.113	0.004	0.094	0.151	0.176	0.173	0.138	0.041	0.123		0.152	0.132	0.184	0.14	0.261	0.402	0.317	0.173
ELB4	0.115	0.098	0.278	0.061	0.213	0.067	0.128	0.142	0.03	0.061	0.000	0.023	0.249	0.075	0.157	0.070	0.008	−0.021	0.09		0.173	0.254	0.192	0.283	0.42	0.345	0.234
ODR1	0.042	0.054	0.253	0.136	0.316*	0.015	0.026	0.06	0.081	0.073	0.044	0.072	0.058	0.132	0.073	0.1	0.117	0.164	0.036	0.153		0.089	0.011	0.361	0.488	0.425	0.187
ODR2	−0.005	−0.001	0.082	0.057	0.191	0.124	0.130	0.172	0.057	0.024	0.112	0.030	0.059	0.048	−0.012	0.023	0.209	0.170	0.17	0.191	0.058		0.116	0.363	0.495	0.429	0.252
ODR3	0.053	0.074	0.277	0.185	0.391*	0.074	0.075	0.133	0.144	0.112	0.129	0.110	0.012	0.186	0.091	0.117	0.227	0.259	0.105	0.263	−0.003	0.050		0.371	0.516	0.437	0.192
ODR4	0.169	0.144	0.263	0.059	0.12	0.261	0.355	0.388*	0.095	0.103	0.185	0.058	0.362	0.084	0.238	0.073	0.197	0.042	0.319*	0.099	0.321	0.267	0.443*		0.148	0.099	0.306
VIS1	0.349	0.34	0.541	0.321	0.468	0.243	0.323	0.324	0.284	0.308	0.223	0.251	0.446	0.336	0.441	0.284	0.175	0.172	0.248	0.140	0.381	0.462	0.502	0.32		0.113	0.425
VIS2	0.261	0.234	0.286	0.14	0.139	0.376*	0.446*	0.478*	0.209	0.194	0.310	0.147	0.432	0.161	0.318	0.124	0.351*	0.169	0.43*	0.239	0.428*	0.341	0.517*	0.079	0.394*		0.334
ITA	0.074	0.077	0.308*	0.121	0.323	0.061	0.018	0.032	0.078	0.080	0.085	0.052	0.11	0.104	0.07	0.077	0.113	0.127	0.104	0.132	0.061	0.092	0.121	0.317	0.373	0.413*	

Pairwise *F*_ST_ values were calculated in GENETIX 4.05 [45], pairwise *R*_ST_ values in SPAGeDi v1.5 [58]. Highlighted values (marked with an asterisk) indicate pairs with non-significant *F*_ST_ values (i.e., *p* > 0.01) and significant differences between pairwise *R*_ST_ and pairwise *pR*_ST_ (i.e., important effect of stepwise mutations on population differentiation)

**Fig. 2:**
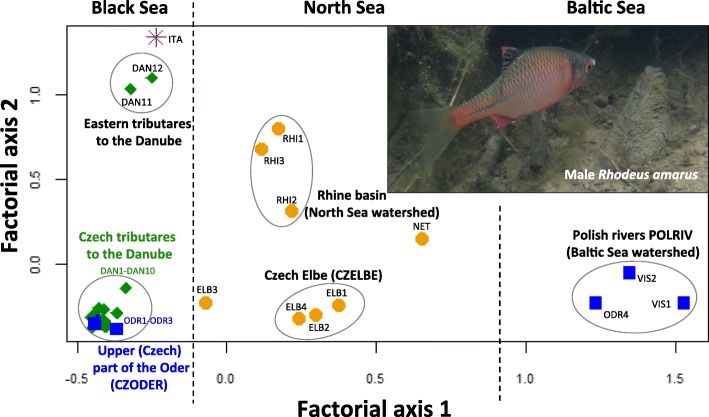
Position of each population based on microsatellite allele frequencies in the Factorial Correspondence Analysis and photograph of male *Rhodeus amarus* with host mussel (inset). Separation by the first factorial axis suggests the existence of a structuring driven by drainage area (illustrated by the hatched lines). The second axis indicates structure driven by variation within individual river basins. Each drainage area is represented by a unique symbol

Factorial correspondence analysis generally separated the three watersheds along the first axis (Fig. 2), but several important exceptions were apparent. The populations from the upper “Czech” part of the River Oder (ODR1-ODR3), belonging to the Baltic watershed, were more similar to populations from the River Morava of the Black Sea watershed than to the Oder population in Poland (ODR4). Population ELB3 from the River Sázava (a tributary of the River Elbe, North Sea watershed) had a position intermediate between populations from the North Sea (the remaining Elbe and Rhine river populations) and Black Sea watersheds (all populations in the Danube basin). An introduced population from the River Po in Italy (ITA) was genetically similar to populations from the River Tisza (DAN11 and DAN12 in eastern Slovakia and Hungary), a major tributary of the River Danube, suggesting its origin from this part of the Black Sea watershed. The second factorial axis sub-structured some specific river basins. The Danube basin populations were separated from the River Tisza populations and the remaining populations in the Czech and Slovak part of this basin. In the North Sea watershed, populations from the River Elbe basin were separated from populations from the River Rhine basin (Fig. 2).

**Fig. 3:**
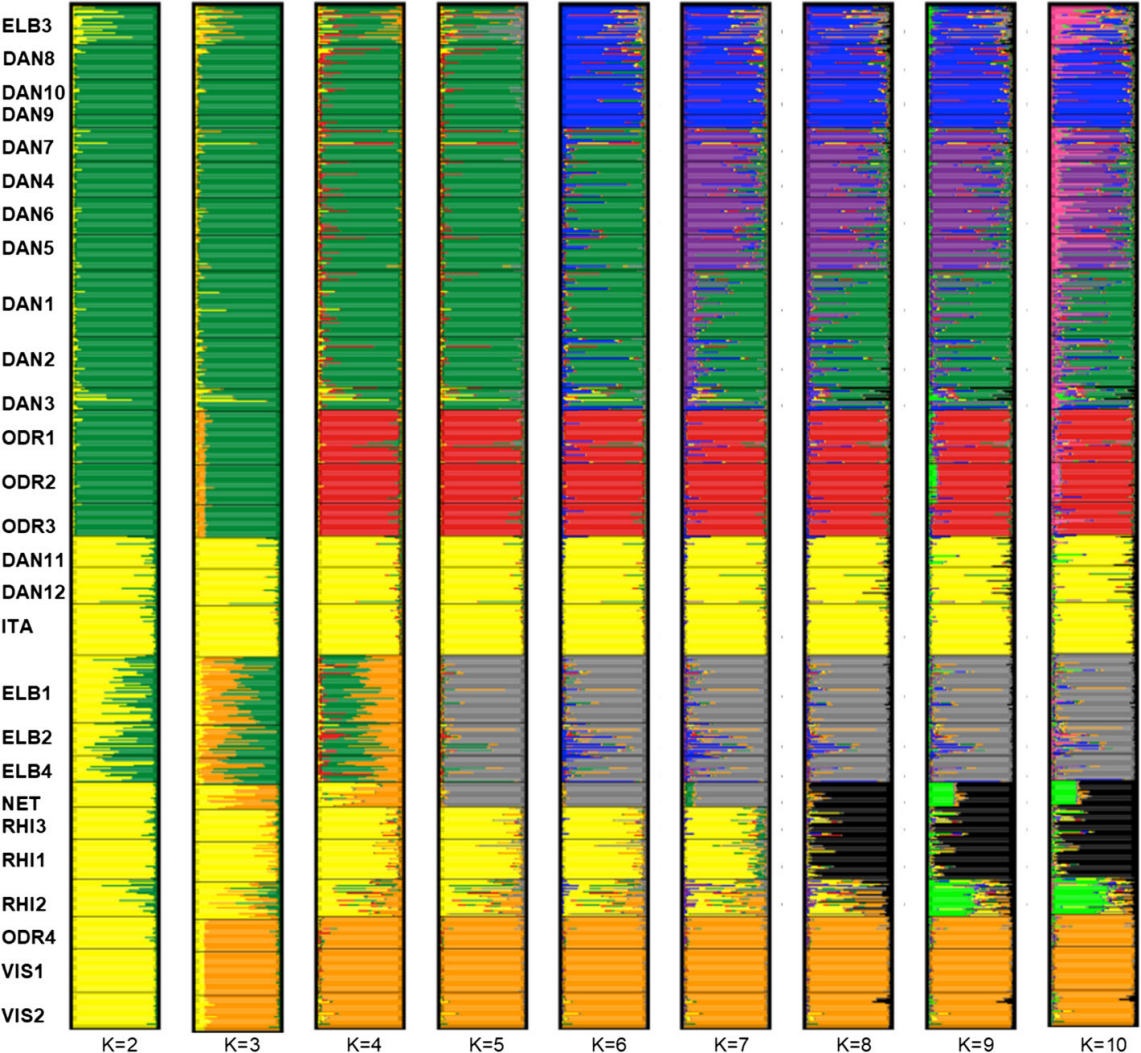
Bayesian analysis of genetic structure of *Rhodeus* populations in central Europe performed in STRUCTURE 2.3.3 [50] for 688 individuals from 27 localities for *K* = 2–10. The results of 10 replicate runs for each value of *K* were combined using the Greedy algorithm of CLUMPP 1.1.2 [52] and summary barplots for each *K*-value were displayed using Distruct v. 1.1 [53]

The best-supported model in STRUCTURE [51] according Δ*K* criterion (Additional file 2) separated all samples into two clusters. This outcome (*K* = 2) is considered a frequent bias of this method [66], but given the presence of two mitochondrial haplotypes, we mapped Q-values for *K* = 2 (Fig. 1d). This provided poor correspondence between watersheds and genetic structure. The same poor correspondence between watersheds and genetic structure was apparent for the specific model assuming clusters corresponding to the three watersheds (i.e., *K* = 3) in central Europe (Additional file 2: Figure S2). Other acceptable models classified populations into 4, 5, 6 and 8 groups; both methods to estimate the best number of clusters (i.e., log-likelihood and Δ*K* criterion) provided a concordant outcome (Additional file 2: Figure S1). Assignments of individuals into particular clusters for *K* = 2–10 are presented in Fig. 3. The comparison of barplots for *K* = 9 and *K* = 10 (Fig. 3) demonstrated that there was no additional meaningful structure in the data. This was further supported by the Δ*K* graphical output (Figure S1b in Additional file 2) showing no support for *K* = 9 and *K* = 10.

A model for *K* = 2 showed that three populations from the River Oder (ODR1-ODR3) located in the Czech Republic clustered with geographically proximate populations from the Danube watershed. The ELB3 population (River Sázava) was more similar to the Danube watershed populations, but was apparently admixed with population from the Elbe (North Sea watershed) and Danube watersheds (Fig. 1d).

**Fig. 4:**
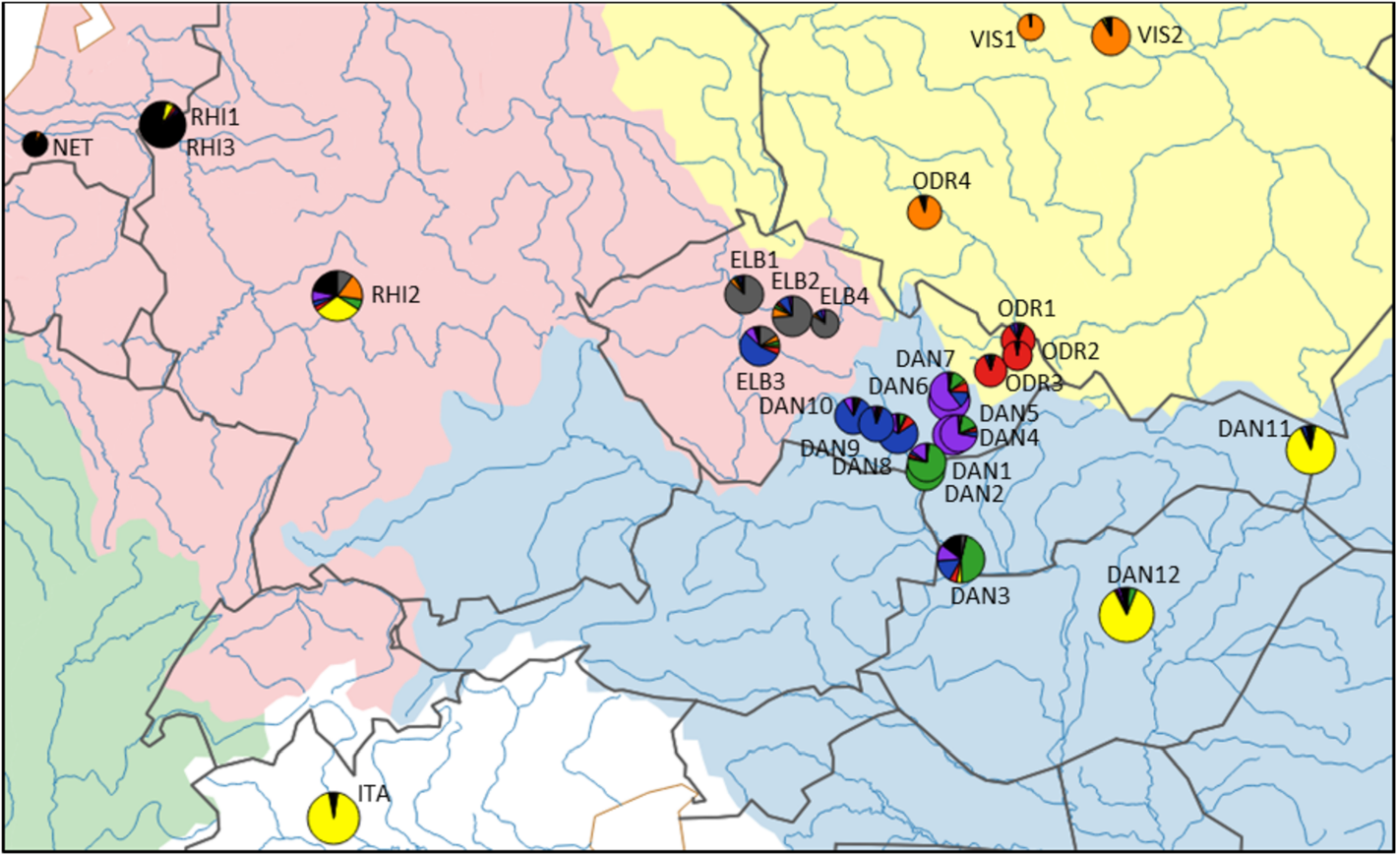
Detailed genetic structure of bitterling populations at the border of three watersheds. Colours of portions in pie charts correspond with the assignment of individuals belonging to a particular group detected in STRUCTURE for *K* = 8. Size of pie charts indicates allelic richness. Hatched lines border groups of populations in the Approximate Bayesian Computation. River basins are illustrated using different colours. The map was created in QGIS 2.18 (http://qgis.org)

A more complex, but suitable (and biologically reliable) model for *K* = 8 revealed significant genetic subdivision in central Europe (Fig. 4) and demonstrated highly heterogeneous populations in the drainages of the River Oder (Baltic watershed) and Elbe (North Sea watershed). First, this analysis confirmed clear separation between the population from the middle part of the River Oder in Poland (ODR4; orange colour in Figs. 3 and 4) and three populations from the upper part of same river (ODR1-ODR3) in the Czech Republic (red colour in Figs. 3 and 4). Second, three populations from the main channel of the Elbe (ELB1, ELB2, ELB4) were similar to each other (grey colour in Figs. 3 and 4), while the population from its southern tributary, the River Sázava (ELB3; blue colour), appeared more closely related to the geographically proximate populations from the Black Sea watershed (DAN8–10).

Populations from the Danube basin (Black Sea watershed) in central Europe demonstrated additional fine-scale substructuring. They were split into three clusters in models for *K* > 7 (Figs. 3 and 4). The first (green colour in Fig. 3 for *K* > 7 and in Fig. 4) is composed of populations from the Slovak stretch of the River Danube and two geographically adjacent tributaries of the Danube at the south-eastern edge of the Czech Republic (the Kyjovka and the lower Morava; DAN1-DAN3). The second cluster (purple) is composed of populations from the middle reaches of the River Morava and its tributaries (DAN4–7). The third cluster (blue) is formed by streams in the River Dyje basin, a major western tributary of the River Morava (DAN8–10).

### The fine-scale contemporary genetic structure among three watersheds

Watersheds were important factors in the genetic structuring of bitterling populations in central Europe. Grouping populations according to watersheds explained a significant proportion (5.7%) of genetic variability (AMOVA: *F*_*CT*_ = 0.057, *p* < 0.001). After removal of the mismatched populations ELB3 and ODR1-ODR3 (see above), the explained component of variation increased to 14% (AMOVA: *F*_*CT*_ = 0.140, *p* < 0.001). This finding indicates that the origin of these mismatched populations was not primarily driven by vicariance among watersheds.

The global *R*_*ST*_ value over all loci was not significantly higher than the mean *pR*_*ST*_, and only 31 of the 351 pairwise comparisons demonstrated significant differences. Focusing on ELB3, none of the three pairwise comparisons between the ELB3 and the remaining Elbe populations (ELB1, ELB2, ELB4) showed significant differences, suggesting that genetic differences within this lineage were caused primarily by genetic drift. On the contrary, one of three pairwise comparisons between the ELB3 population and three geographically proximate Danube populations (DAN8-DAN10) was significant, suggesting some role for stepwise mutations besides genetic drift in population differentiation. The pairwise comparisons among the Czech Oder populations (ODR1-ODR3) did not show any significant differences between *F*_*ST*_ and *R*_*ST*_, and the same was found for the comparison between Czech Oder populations (Baltic Sea) with geographically proximate populations from the River Morava and its tributaries (DAN4-DAN7; Black Sea). On the contrary, 3 out of 9 pairwise *R*_*ST*_ comparisons (33%) between Czech Oder and Polish (ODR4, VIS1, VIS2) populations were significantly higher than *F*_*ST*_ (Table 2), suggesting an important role for mutations in their differentiation and hence an older divergence.

Approximate Bayesian Computation (Additional file 3: Table S1a) suggested that the most likely scenario for the origin of the River Sázava population (SAZAVA) was an admixture between the Elbe (CZELBE, North Sea) and western Morava (WMORAV, Black Sea) groups (posterior probability with a 95% credibility interval: 0.4178, 0.4088–0.4267). The distribution of posterior parameters estimated from this scenario is presented in Additional file 3: Table S3. The WMORAV populations contributed a larger portion of individuals (median: 61.3, 95% CI: 0.129–0.954). The admixture event is dated to approximately 229 years ago (median, 95% CI: 34.4–1020 years ago), followed by a pronounced bottleneck of the effective population size composed of an estimated 69 (median, 95% CI: 13.7–98.7) individuals and lasting approximately 6 years (median, 95% CI: 0–26.2).

The populations from the Czech part of the River Oder (CZODER, Baltic watershed) most likely originated exclusively from the River Danube populations (NMORAV, Black Sea watershed) (posterior probability 0.7451, 95% CI: 0.7387–0.7515) (Additional file 3: Table S1b). Posterior distributions of parameters for this scenario are shown in Additional file 3: Tables S4. The origin of CZODER from NMORAV group (*t3z1*) is estimated to be 130 years ago (median, 95% CI: 25.9–493 years ago), following a bottleneck of an estimated 50 (6.84–97.4) individuals.

## Discussion

### Genetic diversity

The present study supports previous inferences [27, 29] that much of continental Europe was colonised by the bitterling dispersing from two distinct glacial refugia. An eastern clade colonized north-eastern Europe from the Black Sea refugium near the estuaries of the Rivers Dnieper and Dniester. A western clade colonised the rest of Europe from the lower Danube refugium, an important Pleistocene refugium for several other freshwater fish species (e.g. [2, 67, 68]). A secondary contact between these lineages was detected in central Germany (RHI2) in the middle Rhine basin, in accordance with findings for other European freshwater fishes with wide contemporary distributions (e.g. [2, 67, 69]). Low haplotype diversity and a star-like haplotype network for mitochondrial DNA in central and western European bitterling populations supports the interpretation that they expanded into this region within recent centuries, and/or that their population sizes strongly fluctuated during the last 500 years [26].

Genetic diversity was greatest in the Danube basin populations (Black Sea watershed), especially in the Slovakian parts of the Rivers Danube and Tisza. These populations likely retained relatively high genetic variability during recent range contractions and may have served as refugia for subsequent smaller-scale recolonisations. Two other populations were genetically diverse. The bitterling population of the River Po basin in Italy is known to be of recent origin [65]. Its high genetic variability and significant deviation from Hardy-Weinberg equilibrium can be explained by a recent introduction from a genetically highly variable population from the Tisza basin (or repeated introductions from various sources within the Tisza Basin) followed by inbreeding effects or other population genetic processes not yet at equilibrium (Fig. 2, Additional file 2: Figure S2). The River Nida (in the Rhine basin in central Germany) population’s genetic diversity arises from a secondary contact between the two main continental bitterling lineages, with a consequent increase in allelic richness [6].

The lowest genetic diversity in *R. amarus* was typically found in populations at the periphery of the current range (Fig. 3, Additional file 2: Table S1 and Figure S2), with the population from the lower Rhine (NET, Netherlands) being especially depauperate. This finding agrees with the isolation-by-distance scenario whereby variability decreases from a range centre to its periphery, with a significant effect of genetic drift [70]. Two other populations with low genetic diversity were from relatively small standing waters; one from the Czech Oder basin (Pilíky Ponds; ODR2) and one from the River Vistula basin (Lake Kociolek; VIS1) (Table 1).

### Gene flow across the watershed barriers: The role of anthropogenic processes

Bitterling may colonise new habitats via natural dispersal [27, 29] or as a consequence of anthropogenic processes, such as unintentional stocking as a by-product of trade in live fish for cultivation from the 12th to 16th centuries [26] or man-made connections between adjacent drainages [25]. There are good records that the range of European bitterling was extended to many European countries due to intentional introductions over recent decades (e.g., Denmark, Croatia, Italy, Great Britain and the Crimea) [26, 33–35]. It also has been introduced to the Hudson River (New York) in North America in the early twentieth century, where populations persisted despite no evidence of range expansion [71].

In this study, we established that genetic structuring of bitterling in central Europe corresponded well with predictions based on natural dispersal, with a well-defined fine-scale genetic structure within and across river basins. However, there were two notable exceptions. First, the population from the River Sázava (ELB3, Elbe basin, North Sea watershed) was genetically more similar to bitterling populations from the western part of the Black Sea drainage. Second, bitterling populations from the River Oder (Baltic Sea watershed) in the Czech Republic were closely similar to populations from the geographically proximate, but hydrographically disconnected, River Morava basin (Black Sea watershed).

The River Elbe has been recently colonized from the Danube basin by other freshwater fishes, such as *Cottus gobio* [72], *Barbus barbus* [67] and *Cobitis elongatoides* [73], probably as a consequence of human-assisted introductions. Other Black Sea watershed fishes colonized the River Elbe following their escape from aquaculture, such as *Carassius gibelio* [74], or colonising via ballast water, such as *Neogobius melanostomus* [75]. In a previous study [29], we showed that the bitterling population of the River Elbe likely originated from the late-Holocene admixture (dated approximately 1500 years ago) from two main sources - the northeastern (represented here by the Vistula basin and middle Oder population) and upper Danube populations. Here we show that the bitterling population in the River Sázava (tributary of the Elbe) possess a strong signal of much more recent admixture between the Elbe populations (North Sea) and the geographically adjacent populations from the Danube tributaries in western Moravia (Black Sea). The lack of any current physical (natural or artificial) connection between the two neighbouring drainages suggests human activities as the most probable explanation for the observed genetic structure, although small-scale headwater capture events cannot be completely ruled out. Common carp aquaculture has been common in the region since the twelfth century, with potentially frequent transfers of fish (including bitterling) between important aquaculture regions in Moravia and southern Bohemia [26]. The contemporary legal requirement for angling associations to stock commercially available hatchery-reared fish into natural water bodies under their management may also have contributed to repeated recent translocations of freshwater fish populations across drainage boundaries. Such translocations are more relevant for introductions of game fishes, although the bitterling, while of no commercial or angling interest, may be transferred inadvertently with other cyprinids.

The second case of discordance between hydrography and genetic structure includes all three Czech bitterling populations sampled from the River Oder (Baltic Sea drainage). All three populations possessed nuclear and mitochondrial genotypes suggesting their non-admixed origin from the geographically proximate River Morava basin (Black Sea drainage), followed by genetic differentiation by random drift in bottlenecked populations. In contrast, the Polish population from the River Oder clustered with an adjacent population from the River Vistula basin belonging to the same watershed. The large genetic distance between Czech and Polish Oder basin populations pertains at least in part to the inference that mutations, in addition to simple genetic drift, were implicated in the microsatellite marker differences detected between Czech and Polish Oder populations of the bitterling. It is possible that the Czech part of the River Oder basin was not naturally colonised by the bitterling prior to its introduction from the Danube basin, although more than three populations must be examined to test this hypothesis.

### Fragmented and genetically differentiated populations within river basins

The microsatellite analysis revealed profound genetic structuring of bitterling populations on a relatively small geographical scale. Such a high level of genetic differentiation contrasts with the diversity of mitochondrial DNA, with only two lineages evident across continental Europe ([27, 29], this study). Other freshwater fishes with limited dispersal abilities also display strong genetic subdivisions, especially among populations from separate drainages [11, 12]. In general, small cyprinid fishes have a relatively limited capacity for dispersal, resulting in a drainage-restricted intraspecific genetic structure [76] and high species endemism in isolated drainages [31].

Bitterling populations within the Danube basin (Black Sea watershed) demonstrated fine-scale genetic differentiation. The populations were divided into three groups (Fig. 3b), with a clear effect of genetic isolation by geographic distance (isolation-by-distance), with the number of populations studied in this watershed being sufficient for a robust test. The fine structure of the bitterling populations is unlikely a result of river fragmentation by dams. There is a system of three large reservoirs on the River Dyje, isolating western tributaries of the Morava for the last 40–50 years, although no dams are present on the main River Morava or its other tributaries [32]. It is likely that the fine-scale population structure results from events older than the last few decades. Bitterling are recognised as having a limited dispersal capability [77], with downstream drift of the offspring over short distances but limited upstream migration of adult fish prior to spawning [78]. A comparable level of intra-basin differentiation has been recorded in other freshwater fishes [79–81], indicating that fine-scale genetic structuring may be common even in geographically widespread freshwater fishes.

## Conclusions

The genetic structure of the European bitterling in central Europe is affected primarily by the interplay of Quaternary climatic fluctuations and barriers between watersheds. In addition, we detected anthropogenic effects on the genetic variability of some bitterling populations, with one population admixed from two different watersheds and a group of geographically proximate populations misclassified from expectation on the basis of hydrography. Given the lack of natural or artificial connections between adjacent drainages, dispersal across watersheds likely involved intentional or unintentional stocking in the course of fish cultivation or as an effect consequent of angling. Local translocations within a species’ range can cause spread of non-native lineages outside their former range. It becomes apparent that such cryptic invasions are more widespread than commonly recognized [17], with apparent consequences for conservation management of local communities [82]. Despite introductions in the specific cases, contemporary bitterling populations are principally structured by their respective drainage, including fine-scale differentiation through isolation by distance. As such, genetic structure of bitterling populations in central Europe is predominantly natural, despite introductions of the species in peripheral regions of Europe. Finally, our study demonstrated that studies of species with no commercial value may reveal how historical range dynamics are mirrored by contemporary genetic diversity within and among populations, and exhibit patterns indicating human-assisted colonisation.

## Additional files

Additional file 1: Protocols for genotyping microsatellites and mtDNA. (DOCX 1965 kb)

Additional file 2: Additions to genetic analyses. **Figure S1.** Evaluation of 10 runs in STRUCTURE 2.3.3 [3] for each number of inferred clusters from *K* = 2 to *K* = 10. (a) Likelihood (ln Pr(*X*|*K*)) of models in STRUCTURE for increasing number of hypothetical populations (*K*); (b) Estimation of the best *K* division using the Δ*K* criterion of Evanno et al. [51]. **Figure S2.** Detailed genetic structure of the bitterling populations in central Europe. For *K* = 3 and *K* = 8. (http://qgis.org). **Table S1.** Genetic variability of populations. Genetic variability was assessed by 12 and 9 loci. (DOCX 294 kb)

Additional file 3: Approximate Bayesian Computation analyses. **Table S1.** Summary of scenarios used in the approximate Bayesian computation analysis to infer the origin of the Sázava river population and three Czech Oder basin populations. **Table S2.** The prior parameter distributions used in five scenarios for the origin of SAZAVA origin in DIYABC 2.0.4. **Table S3.** The posterior parameter distributions of SAZAVA population. **Table S4.** The posterior parameter distributions of CZODER population. **Figure S1.** Graphical schemes of scenarios used for analysis in DIYABC 2.0.4 to infer of origin of the SAZAVA population. **Figure S2.** Graphical schemes of scenarios used for analysis in DIYABC 2.0.4 to infer of origin of the CZODER population. (DOCX 29 kb)

## Abbreviations

AMOVA: Analysis of Molecular Variance
*AR*: Allelic richness
*CYTB*: The gene for cytochrome *b*
FCA: Factorial correspondence analysis
GSM: Generalized stepwise model
*HWE*: Hardy-Weinberg equilibrium
