# Supplementary material for: Fine-scale genetic structure of the European bitterling at the intersection of three major European watersheds
Source: BMC Evol Biol. 2018 Jul 4;18:105. doi: 10.1186/s12862-018-1219-9 (PMC6030748; doi:10.1186/s12862-018-1219-9)
Supplement: Supplementary file 1 — Protocols for genotyping microsatellites and mtDNA. (DOCX 1965 kb) [file 12862_2018_1219_MOESM1_ESM.docx]

**Additional file 1** Protocols for genotyping of microsatellites and mtDNA

***(1) DNA extraction and genotyping of microsatellite loci***

DNA extraction was performed using the JETQUICK Tissue DNA Spin Kit (Genomed; <http://www.genomed-dna.com/>). Extracted DNA was stored at -20°C. All bitterling individuals were genotyped at 12 microsatellite loci in three multiplex PCR sets (Tables S1 and S2), using a modified protocol from Bryja et al. [1]. The reaction mix contained 1 μL extracted DNA, 0.2 mM dNTPs, 1.2 mM MgCl2, 0.2 U Taq polymerase (Fermentas), 1x buffer, primers in various concentration (Table S1) and ddH_2_O up to the total volume 10 μL. The amplification took place in thermocycler Mastercycler ep gradient S (Eppendorf).

Temperature profile of PCR for microsatellites:

94 °C (3 min) - 1 cycle

94 °C (30 s) 61 °C (30 s) 72°C (40 s) - 30 cycles

72 °C (5 min) - 1 cycle

The mix for fragment analysis was composed of 1 μL of PCR product, 12 μl of formamide and 0.3 μl of size standard (ROX 500, Applied Biosystems). After 5 min denaturation at 96 °C and 2 min cooling on ice, the capillary electrophoresis was run on the ABI Prism® 3130 Genetic Analyzer (Applied Biosystems). The length of DNA fragments was analysed manually using GeneMapper v. 3.7 (Applied Biosystems).

**Table S1 Overview of genotyped microsatellite loci.**

| Marker ID | | Label | µM | Set | A | NA(%) |
| --- | --- | --- | --- | --- | --- | --- |
| Rser01 | A08 | FAM | 0.15 | 2 | 7 | 1 |
| Rser02 | A10 | NED | 0.125 | 1 | 9 | 2.6 |
| Rser03 | C05 | HEX | 0.1 | 1 | 6 | 2.7 |
| Rser04* | F07* | NED | 0.2 | 1 | 94 | 6.9 |
| Rser05 | F01 | FAM | 0.2 | 2 | 7 | 2.3 |
| Rser06 | G04 | FAM | 0.15 | 2 | 19 | 1.4 |
| Rser08 | D04 | HEX | 0.175 | 1 | 14 | 0.7 |
| Rser09* | D12* | NED | 0.15 | 3 | 26 | 7.9 |
| Rser10 | E01 | FAM | 0.1 | 2 | 14 | 0.8 |
| Rser11 | G01 | HEX | 0.25 | 2 | 30 | 1.1 |
| Rser12 | H04 | HEX | 0.2 | 3 | 7 | 3.4 |
| Rser13* | D05* | FAM | 0.15 | 3 | 95 | 8.2 |

** problematic loci excluded from some analyzes due to the high frequency of null alleles and/or incomparably high number of alleles (see the text for more details)*

*Label:* fluorescent label of loci

*µM*: final concentration of each forward and reverse primer in a multiplex PCR

*Set*: number of multiplex PCR set

*A*: number of alleles

*NA(%):* mean frequencies of null alleles per population estimated in FreeNA [4]

**Table S2: Primer sequences of 12 polymorphic bitterling microsatellite loci.**

| **Marker ID** | | **Forward Oligo Sequence** | **Reverse Oligo Sequence** | **Reference** |
| --- | --- | --- | --- | --- |
| Rser01 | A08 | GACAGCGGTGAAAGTCACTTATGC | ATCCTCCGCTAGTGAGCGCC | 5 |
| Rser02 | A10 | GATCTGCACCTCAGGCAAGC | AGGACGCCCATTCTGATGC | 5 |
| Rser03 | C05 | TGACAGGCAGGAAACAGCCA | CAGTGCAACTCTTCCATTATGTCCC | 5 |
| Rser04 | F07 | GGCACAGAAGTTGGGTGTGC | GCTATCGCCACGGACCATTG | 5 |
| Rser05 | F01 | AACGCTCAAGCTCACAATCA | AGCCCTACTTGAGCCATTGA | 5 |
| Rser06 | G04 | CGGGTACTGTAGTGGCGTGG | CCTTCTGCACGTACATCATCAGC | 5 |
| Rser08 | D04 | CGCAGCTGTCAGCAAGACTG | ACTGAAGCGTCCTCCATCGC | 5 |
| Rser09 | D12 | CGGGCGAGTCGAACATAAATTG | TGCCATAAAGCTCCGCTTCAC | 5 |
| Rser10 | E01 | TGCGTAATCGTGAAGCGGTG | GCCACTAAAGCGCAGAAGCC | 5 |
| Rser11 | G01 | AATGTGCTTTGAATGTCACCTAA | CCAAGCTGAAGTTGAAACAAAC | 5 |
| Rser12 | H04 | CATCTGGTGGTTTGCATGTC | TTTAGCCGTCTCTCAGCCTC | 5 |
| Rser13 | D05 | GCATACTGTTAAGCCACCCGC | AGCATTGGCAAGGTGGGAGAG | 6 |

***(2) Genotyping of mtDNA***

Mitochondrial gene for cytochrome *b* (*CYTB*) was genotyped in a subset of individuals (see Table 1 in the main text). Partial sequence (1124 bp) was amplified by primers Thr-H (5´-ACCTCCRATCTYCGGATTACA-3´) and Glu-L (5´-GAAGAACCACCGTTGTTATTCAA-3´) following the protocol of Bohlen et al. [2]. The reaction mix contained 3 μL extracted DNA, 15 μL Multiplex PCR kit (Qiagen), primers in final concentration 1.5 μM and ddH_2_O up to the total volume 30 μL.

Temperature profile of PCR for cytochrome *b:*

95 °C (15 min) - 1 cycle

94 °C (30 sec) 50 °C (90 sec) 72 °C (90 ses) - 30 cycles

72 °C (10 min) - 1 cycle

PCR products were commercially Sanger-sequenced in Macrogen Europe. Sequence editing was performed in SeqScape V.2.5 (Applied Biosystems) and BioEdit v.7.0.9.0 [3].

***References cited:***

1. Bryja J, Smith C, Konečný A, Reichard M. Range-wide population genetic structure of the European bitterling (*Rhodeus amarus*) based on microsatellite and mitochondrial DNA analysis. Mol Ecol. 2010;19:4708–4722.
2. Bohlen J, Šlechtová V, Bogutskaya N, Freyhof J. Across Siberia and over Europe: Phylogenetic relationships of the freshwater fish genus *Rhodeus* in Europe and the phylogenetic position of *R. sericeus* from the River Amur. Mol Phylogenet Evol. 2006;40:856–865.
3. Hall TA. BioEdit: a user-friendly biological sequence alignment editor and analysis program for Windows 95/98/NT. Nucleic Acids Symp Ser (Oxf). 1999;41:95–98.
4. Chapuis MP, Estoup A. Microsatellite null alleles and estimation of population differentiation. Mol Biol Evol. 2007;24:621–631.
5. Dawson DA, Burland TM, Douglas A, Le Comber SC, Bradshaw M. Isolation of microsatellite loci in the freshwater fish, the bitterling *Rhodeus sericeus* (Teleostei: Cyprinidae). Mol Ecol Notes. 2003;3:199–202.
6. Reichard M, Smith C, Bryja J. Seasonal change in the opportunity for sexual selection. Mol Ecol. 2008;17:642–651.
