# Supplementary material for: Fine-scale genetic structure of the European bitterling at the intersection of three major European watersheds
Source: BMC Evol Biol. 2018 Jul 4;18:105. doi: 10.1186/s12862-018-1219-9 (PMC6030748; doi:10.1186/s12862-018-1219-9)
Supplement: Supplementary file 2 — Additions to genetic analyses. Figure S1. Evaluation of 10 runs in STRUCTURE 2.3.3 [3] for each number of inferred clusters from K = 2 to K = 10. (a) Likelihood (ln Pr(X|K)) of models in STRUCTURE for increasing number of hypothetical populations (K); (b) Estimation of the best K division using the ΔK criterion of Evanno et al. [51]. Figure S2. Detailed genetic structure of the bitterling populations in central Europe. For K = 3 and K = 8. (http://qgis.org). Table S1. Genetic variability of populations. Genetic variability was assessed by 12 and 9 loci. (DOCX 294 kb) [file 12862_2018_1219_MOESM2_ESM.docx]

**Additional File 2** Supplementary tables and figures with details on genetic analyses

**Figure S1** Evaluation of 10 runs in STRUCTURE 2.3.3 [3] for each number of presumable clusters from *K*=2 to *K*=10. (a) Likelihood (ln Pr(X|K)) of models in STRUCTURE for increasing number of hypothetical populations (*K*); (b) Estimation of the best *K* division using the ΔK criterion according to Evanno et al. [4]. The values indicate relative increase of credibility depending on the number of *K*.

(a)


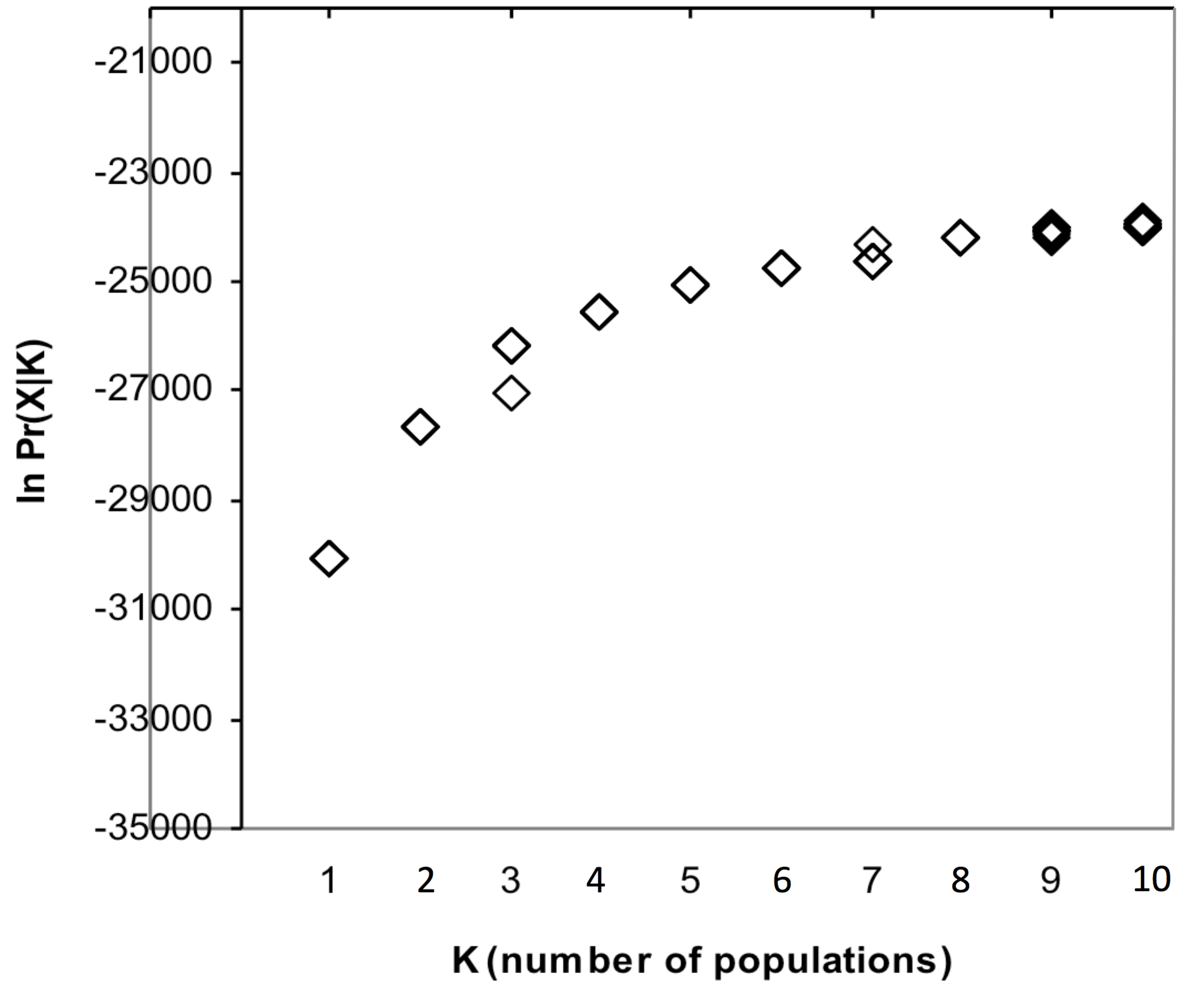


(b)

**
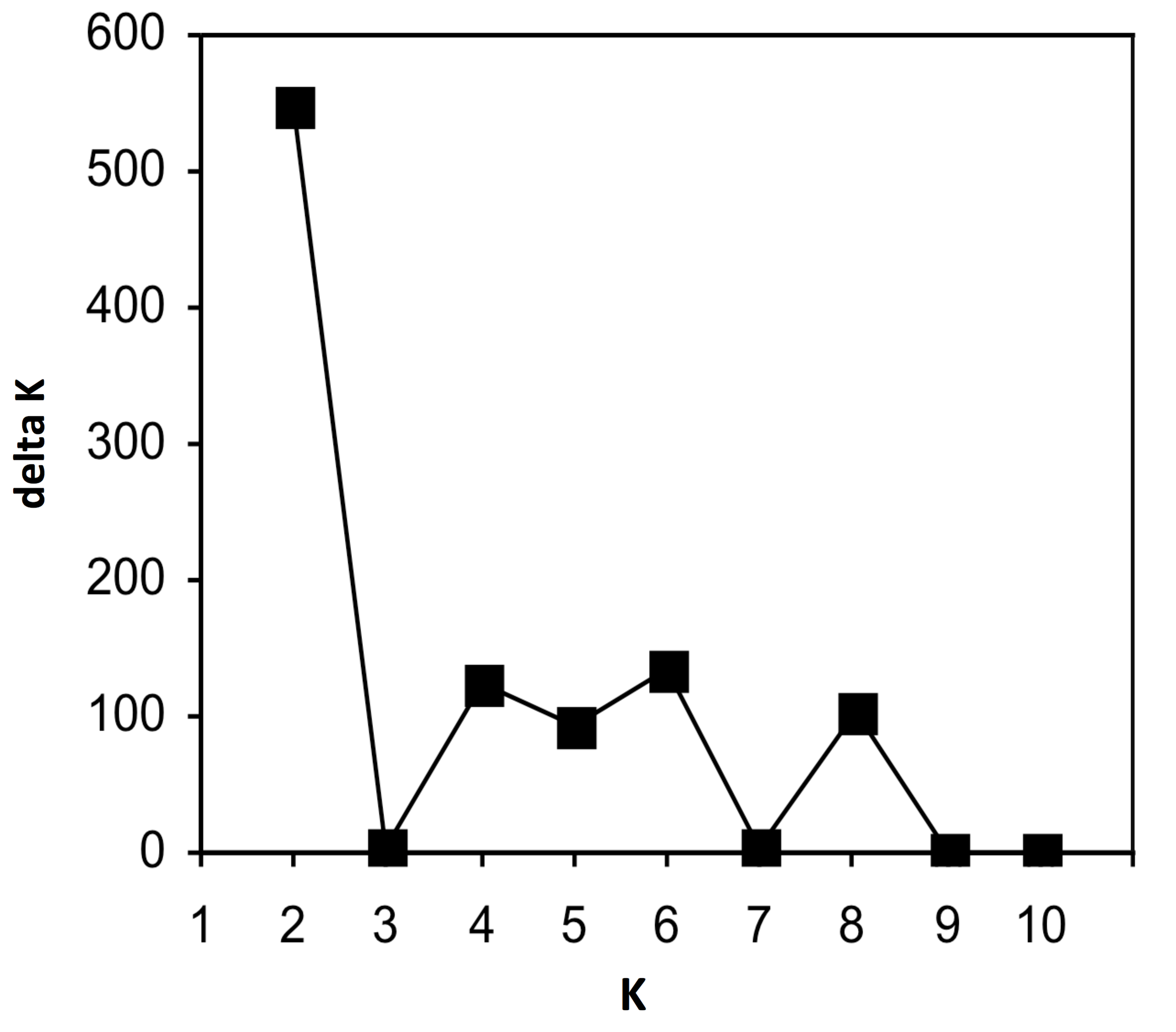
**

**Figure S2** Detailed genetic structure of bitterling populations in central Europe. The colours of pie charts correspond to population *Q*-values; i.e. probability of belonging to a particular population cluster detected in STRUCTURE 2.3.3 [3] for K = 3 (upper figure). Diameter of pie charts corresponds to allelic richness of particular populations. The map was created in QGIS 2.18 (http://qgis.org).

**
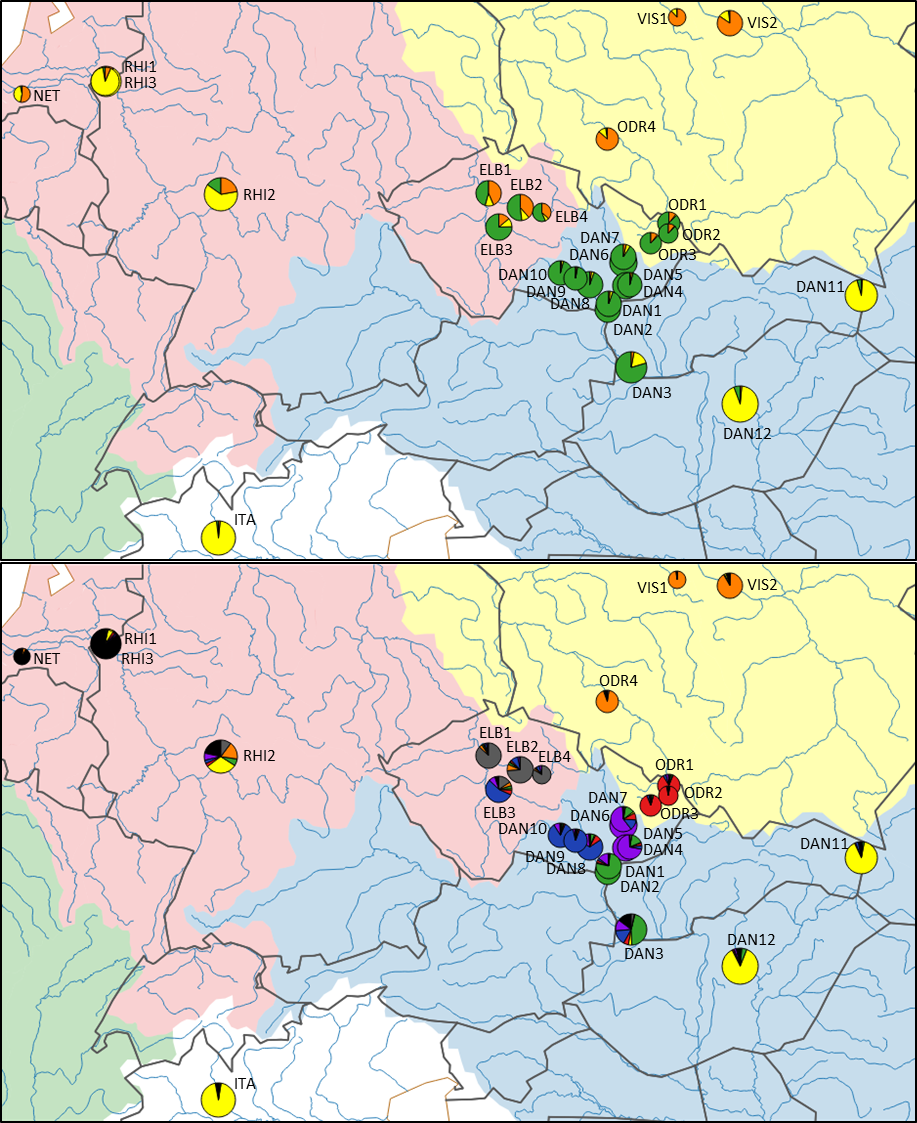
**

**Table S1** Genetic variability of populations

| Locality | *He*(12) | *Ho*(12) | *He*(9) | *Ho*(9) | *AR*(12) | *AR*(9) | *HWE*(12) | *HWE*(9) | Provider |
| --- | --- | --- | --- | --- | --- | --- | --- | --- | --- |
| DAN1 | 0.59 | 0.56 | 0.50 | 0.47 | 5.01 | 3.51 | 0.276 | 0.220 | M. Reichard |
| DAN2 | 0.56 | 0.57 | 0.46 | 0.49 | 5.09 | 3.52 | 0.528 | 0.973 | M. Ondračková |
| DAN3 | 0.65 | 0.60 | 0.58 | 0.57 | 5.85 | 4.31 | **<0.001** | 0.398 | M. Ondračková |
| DAN4 | 0.61 | 0.57 | 0.52 | 0.54 | 5.16 | 3.72 | **<0.001** | 0.217 | Z. Valová et al. |
| DAN5 | 0.56 | 0.50 | 0.47 | 0.46 | 4.83 | 3.41 | **<0.001** | 0.648 | Z. Valová et al. |
| DAN6 | 0.61 | 0.58 | 0.55 | 0.56 | 5.07 | 3.86 | **<0.001** | 0.630 | Z. Valová et al. |
| DAN7 | 0.55 | 0.48 | 0.44 | 0.42 | 5.09 | 3.56 | **<0.001** | 0.561 | Z. Valová et al. |
| DAN8 | 0.59 | 0.54 | 0.52 | 0.50 | 4.57 | 3.65 | 0.114 | 0.694 | Z. Valová et al. |
| DAN9 | 0.53 | 0.55 | 0.45 | 0.48 | 4.42 | 3.33 | 0.791 | 0.623 | Z. Valová et al. |
| DAN10 | 0.54 | 0.53 | 0.44 | 0.45 | 4.57 | 3.35 | 0.295 | 0.933 | Z. Valová et al. |
| DAN11 | 0.65 | 0.61 | 0.56 | 0.54 | 6.46 | 4.49 | 0.286 | 0.552 | J. Bohlen |
| DAN12 | 0.71 | 0.69 | 0.64 | 0.65 | 6.95 | 5.05 | 0.320 | 0.963 | T. Eros |
| RHI1 | 0.74 | 0.69 | 0.68 | 0.65 | 5.98 | 4.27 | **0.016** | 0.177 | J. Bohlen |
| RHI2 | 0.75 | 0.63 | 0.70 | 0.69 | 5.76 | 4.57 | **<0.001** | 0.316 | K. Schindehütte |
| RHI3 | 0.72 | 0.63 | 0.66 | 0.66 | 5.68 | 3.96 | **<0.001** | 0.267 | M. Ondračková |
| NET | 0.52 | 0.53 | 0.47 | 0.47 | 3.06 | 2.33 | 0.685 | 0.706 | M. Soes |
| ELB1 | 0.62 | 0.62 | 0.56 | 0.56 | 4.90 | 3.55 | 0.484 | 0.654 | M. Ondračková |
| ELB2 | 0.63 | 0.52 | 0.56 | 0.52 | 4.76 | 3.66 | **<0.001** | 0.338 | Z. Valová et al. |
| ELB3 | 0.62 | 0.57 | 0.54 | 0.53 | 5.11 | 3.63 | **<0.001** | 0.658 | P. Horký |
| ELB4 | 0.50 | 0.48 | 0.41 | 0.42 | 3.73 | 2.64 | 0.738 | 0.989 | M. Ondračková |
| ODR1 | 0.56 | 0.51 | 0.46 | 0.47 | 4.42 | 3.11 | **<0.001** | 0.270 | Z. Valová et al. |
| ODR2 | 0.53 | 0.53 | 0.49 | 0.49 | 3.32 | 2.68 | 0.355 | 0.348 | Z. Valová et al. |
| ODR3 | 0.55 | 0.47 | 0.46 | 0.40 | 4.01 | 2.98 | **0.026** | 0.189 | M. Ondračková |
| ODR4 | 0.49 | 0.49 | 0.45 | 0.47 | 3.78 | 3.12 | 0.491 | 0.977 | M. Ondračková |
| VIS1 | 0.30 | 0.30 | 0.29 | 0.29 | 2.93 | 2.46 | 0.998 | 1.000 | M. Przybylski |
| VIS2 | 0.44 | 0.42 | 0.39 | 0.36 | 4.28 | 3.48 | 0.121 | 0.214 | M. Ondračková |
| ITA | 0.72 | 0.58 | 0.65 | 0.58 | 6.09 | 4.68 | **<0.001** | **<0.001** | M. Ondračková |

Genetic variability was assessed by 12 and 9 loci (number in bracket). H_e_ = mean expected heterozygosity, *H*_o_ = mean observed heterozygosity, *AR* = allelic richness standardized on sample size, *HWE* = P-values of Hardy-Weinberg Equilibrium tests (*HWE*). Highlighted values indicate population deviating from *HWE*.

***References cited:***

1. Belkhir K, Borsa P, Chikhi L, Raufaste N, Bonhomme F. GENETIX 4.05, logiciel sous Windows TM pour la génétique des populations. Laboratoire Génome, Populations, Interactions, CNRS UMR. 1996;5000:1996–2004.
2. Hardy OJ, Vekemans X. spagedi: a versatile computer program to analyse spatial genetic structure at the individual or population levels. Mol Ecol Notes. 2002;2:618–620.
3. Falush D, Stephens M, Pritchard JK. Inference of population structure using multilocus genotype data: linked loci and correlated allele frequencies. Genetics. 2003;164:1567–1587.
4. Evanno G, Regnaut S, Goudet J. Detecting the number of clusters of individuals using the software STRUCTURE: a simulation study. Mol Ecol. 2005;14:2611–2620.
5. Jakobsson M, Rosenberg NA. CLUMPP: a cluster matching and permutation program for dealing with label switching and multimodality in analysis of population structure. Bioinformatics. 2007;23:1801–1806.
6. Rosenberg NA. Distruct: a program for the graphical display of population structure. Mol Ecol Notes. 2004;4:137–138.
