# Supplementary material for: Fine-scale genetic structure of the European bitterling at the intersection of three major European watersheds
Source: BMC Evol Biol. 2018 Jul 4;18:105. doi: 10.1186/s12862-018-1219-9 (PMC6030748; doi:10.1186/s12862-018-1219-9)
Supplement: Supplementary file 3 — Approximate Bayesian Computation analyses. Table S1. Summary of scenarios used in the approximate Bayesian computation analysis to infer the origin of the Sázava river population and three Czech Oder basin populations. Table S2. The prior parameter distributions used in five scenarios for the origin of SAZAVA origin in DIYABC 2.0.4. Table S3. The posterior parameter distributions of SAZAVA population. Table S4. The posterior parameter distributions of CZODER population. Figure S1. Graphical schemes of scenarios used for analysis in DIYABC 2.0.4 to infer of origin of the SAZAVA population. Figure S2. Graphical schemes of scenarios used for analysis in DIYABC 2.0.4 to infer of origin of the CZODER population. (DOCX 29 kb) [file 12862_2018_1219_MOESM3_ESM.docx]

**Additional File 3** Approximate Bayesian Computation analyses

**Table S1** Summary of scenarios used in the approximate Bayesian computation analysis (ABC) [1] to infer the origin of the Sázava river population (A) and three Czech Oder basin populations (B)

| (A) Sázava | Description of scenario | Posterior probability | Credibility interval |
| --- | --- | --- | --- |
| Scenario 1 | From *AN* | 0.2780 | [0.2697,0.2862] |
| Scenario 2 | From CZELBE | 0.0653 | [0.0600,0.0705] |
| Scenario 3 | From WMORAV | 0.2149 | [0.2076,0.2223] |
| **Scenario 4** | **Mix CZELBE and WMORAV** | **0.4178** | **[0.4088,0.4267]** |
| Scenario 5 | From NG | 0.0241 | [0.0193,0.0288] |

| (B) Czech Oder | Description of scenario | Posterior probability | Credibility interval |
| --- | --- | --- | --- |
| Scenario 1 | From AN | 0.0496 | [0.0466,0.0527] |
| **Scenario 2** | **From NMORAV** | **0.7451** | **[0.7387,0.7515]** |
| Scenario 3 | Mix POLRIV and NMORAV | 0.1934 | [0.1874,0.1994] |
| Scenario 4 | From NG | 0.0119 | [0.0101,0.0137] |

The analyses were computed in DIYABC 2.0.4 [2]. The most supported scenario is indicated in bold typeset. The relative posterior probability (95% confidence intervals) of each scenario was computed via the logistic regression with the 1% (n = 50 000 and 40 000, respectively) of the simulated data sets closest to the observed data. SAZAVA = ELB3; CZELBE = ELB1 + ELB2 + ELB4; WMORAV = DAN8-DAN10; CZODER = ODR1-ODR3; NMORAV = DAN1 + DAN2 + DAN4-DAN7; POLRIV = ODR4 + VIS1 + VIS2; *AN*: a hypothetical ancestral population; NG: ghost, unsampled population.

**Table S2** The prior parameter distributions used in five scenarios for the origin of SAZAVA origin in DIYABC 2.0.4 [2].

| Parameter | Distribution | Min. | Max. |
| --- | --- | --- | --- |
| **Effective population size** |  |  |  |
| *N1, N2, N3, N0, NG* | uniform | 10 | 5000 |
| *nf3* | uniform | 2 | 100 |
| **Time of events** |  |  |  |
| *t1, t2, t3, t3z2, t3z1, tx, t3zg, tg* | uniform | 10 | 5000 |
| *db3* | uniform | 0 | 30 |
| **Admixture rate** |  |  |  |
| *r* | uniform | 0.001 | 0.999 |
| **Mutation model parameters** |  |  |  |
| Mean mutation rate | uniform | 0.0001 | 0.001 |
| Mean coefficient *p* | uniform | 0.1 | 0.3 |

In four scenarios for the origin of the CZODER populations we used the same prior parameters and their distributions, except parameter *t3z2*. We also used following assumptions: *t3z2*<*t2*, *db3*<*t3z2*, *t3z1*<1, *tx*<t2, *tx*<*t1*, *db3*<*tx*, *tg*>*t3zg*, *db3*<*t3z1*, *db3*<*t3zg*, *nf3*<=*N3*. The first two assumptions were omitted in tests of CZODER origin. See also Figures S1 and S2, especially for the abbreviations of time of events.

**Table S3** The posterior parameter distributions of SAZAVA population

| Parameter | Mean | Median | Mode | 95% CI |
| --- | --- | --- | --- | --- |
| WMORAV | 3230 | 3260 | 3090 | 1260 - 4870 |
| CZELBE | 3340 | 3380 | 3280 | 1450 - 4880 |
| SAZAVA | 3330 | 3430 | 3900 | 1110 - 4920 |
| *AN* | 1770 | 1520 | 622 | 108 - 4570 |
| *nf3* | 65.2 | 68.6 | 99.1 | 13.7 - 98.7 |
| *t1* | 1260 | 960 | 472 | 147 - 3970 |
| *t2* | 1110 | 816 | 443 | 120 - 3880 |
| *tx* | 303 | 229 | 110 | 34.4 - 1020 |
| *db3* | 8.17 | 6.18 | 0 | 0 - 26.2 |
| *r* | 0.598 | 0.613 | 0.605 | 0.129 - 0.954 |
| Mean mutation rate | 0.000334 | 0.000301 | 0.000260 | 0.000132 - 0.000733 |
| Mean coefficient p | 0.231 | 0.238 | 0.3 | 0.125 - 0.3 |

The posterior parameter distributions were estimated using the Approximate Bayesian Computation (ABC, [1]) from the closest 1% simulated data of the most likely scenario for the origin of SAZAVA. WMORAV, CZELBE, SAZAVA, *AN* = effective population sizes of particular populations; *nf3* = the effective population size of the SAZAVA population during the duration of bottleneck *db3* (i.e. number of founder individuals of the SAZAVA population); *t1* and *t2* = times of divergence of WMORAV and CZELBE populations from the ancestral population (*AN*); *r* = rate of admixture; i.e. the proportion of the population WMORAV in the time of SAZAVA origin. Time parameters are in generations (= years, because we used a generation time of 1 year) backwards in time. Mean coefficient p = marker parameter of the geometric distribution of the length in number of repeats of mutation events (under the GSM).

**Table S4** The posterior parameter distributions of CZODER population.

| Parameter | Mean | Median | Mode | 95% CI |
| --- | --- | --- | --- | --- |
| NMORAV | 3640 | 3720 | 3970 | 1960 - 4880 |
| POLRIV | 3500 | 3580 | 3730 | 1740 - 4860 |
| CZODER | 1880 | 1640 | 946 | 386 - 4560 |
| *AN* | 1120 | 726 | 151 | 57.9 - 4160 |
| *nf3* | 50.8 | 49.6 | 23.4 | 6.84 - 97.4 |
| *t1* | 2940 | 3040 | 4340 | 478 - 4910 |
| *t2* | 2330 | 2250 | 329 | 114 - 4840 |
| *t3z1* | 164 | 130 | 75.7 | 25.9 - 493 |
| *db3* | 5.54 | 5.74 | 10 | 0 - 10 |
| Mean mutation rate | 0.000365 | 0.000333 | 0.000239 | 0.000134 - 0.000775 |
| Mean coefficient p | 0.259 | 0.270 | 0.3 | 0.153 - 0.3 |

The posterior parameter distributions estimated via the Approximate Bayesian Computation (ABC, [1]) from the closest 1% simulated data of the most likely scenario for the origin of CZODER population. NMORAV, POLRIV, CZODER, AN = effective population sizes of particular populations; *nf3* = number of founder individuals of the CZODER population, *db3* = duration of bottleneck from the event of divergence the CZODER population from the NMORAV population in time *t3z1*; *t1* and *t2* = times of divergence of NMORAV and POLRIV populations from the ancestral population (*AN*). Time parameters are in years backward in time.

**Figure S1** Graphical schemes of scenarios used for analysis in DIYABC 2.0.4 [2] to infer of origin of the SAZAVA population. The most likely scenario was determined Scenario 4, for numerical results see Table 2a in the main text.

**Figure S2** Graphical schemes of scenarios used for analysis in DIYABC 2.0.4 [2] to infer of origin of the CZODER population. The most likely scenario was determined Scenario 2, for numerical results see Table 2b in the main text.

***References cited:***

1. Beaumont MA, Zhang W, Balding DJ. Approximate Bayesian Computation in Population Genetics. Genetics. 2002;162:2025–2035.
2. Cornuet JM, Pudlo P, Veyssier J, Dehne-Garcia A, Gautier M, Leblois R, Marin JM, Estoup A. DIYABC v2.0: a software to make Approximate Bayesian Computation inferences about population history using Single Nucleotide Polymorphism, DNA sequence and microsatellite data. Bioinformatics. 2014;30:1187–1189.
